# Selective Essential Oils from Spice or Culinary Herbs Have High Activity against Stationary Phase and Biofilm *Borrelia burgdorferi*

**DOI:** 10.3389/fmed.2017.00169

**Published:** 2017-10-11

**Authors:** Jie Feng, Shuo Zhang, Wanliang Shi, Nevena Zubcevik, Judith Miklossy, Ying Zhang

**Affiliations:** ^1^Department of Molecular Microbiology and Immunology, Bloomberg School of Public Health, Johns Hopkins University, Baltimore, MD, United States; ^2^Department of Physical Medicine and Rehabilitation, Harvard Medical School, Spaulding Rehabilitation Hospital, Charlestown, MA, United States; ^3^International Alzheimer Research Centre, Prevention Alzheimer International Foundation, Martigny-Croix, Switzerland

**Keywords:** *Borrelia burgdorferi*, persisters, biofilm, essential oils, carvacrol

## Abstract

Although the majority of patients with acute Lyme disease can be cured with the standard 2–4 week antibiotic treatment, about 10–20% of patients continue suffering from chronic symptoms described as posttreatment Lyme disease syndrome. While the cause for this is debated, one possibility is that persister bacteria are not killed by the current Lyme antibiotics and remain active in the system. It has been reported that essential oils have antimicrobial activities and some have been used by patients with persisting Lyme disease symptoms. However, the activity of essential oils against the causative agent *Borrelia burgdorferi* (*B. burgdorferi*) has not been well studied. Here, we evaluated the activity of 34 essential oils against *B. burgdorferi* stationary phase culture as a model for persister bacteria. We found that not all essential oils had activity against the *B. burgdorferi* stationary phase culture, with top five essential oils (oregano, cinnamon bark, clove bud, citronella, and wintergreen) at a low concentration of 0.25% showing high anti-persister activity that is more active than the known persister drug daptomycin. Interestingly, some highly active essential oils were found to have excellent anti-biofilm ability as shown by their ability to dissolve the aggregated biofilm-like structures. The top three hits, oregano, cinnamon bark, and clove bud completely eradicated all viable cells without any regrowth in subculture in fresh medium, whereas but not citronella and wintergreen did not have this effect. Carvacrol was found to be the most active ingredient of oregano oil showing excellent activity against *B. burgdorferi* stationary phase cells, while other ingredients of oregano oil p-cymene and α-terpinene had no apparent activity. Future studies are needed to characterize and optimize the active essential oils in drug combination studies *in vitro* and *in vivo* and to address their safety and pharmacokinetic properties before they can be considered as a novel treatment of persistent Lyme disease.

## Introduction

Lyme disease or Borreliosis is the most common vector borne illness in the United States with an estimated 300,000 cases per year (1). The illness is transmitted by a tick bite and in some endemic areas, where more than 40% of the ticks are infected with the causative agent of Borreliosis, *Borrelia burgdorferi* sensu lato complex species, which increases the risk of transmission to human host (2).

The difficulty in the clinical management of Borreliosis is that the current treatment regimen recommended for acute stage of illness of 21 days of Doxycycline (Dox) (3) leaves over 20% of patients with chronic symptoms that can last over 6 months (4). These chronic symptoms can be debilitating fatigue, muscular and joint pain, and cognitive and neurologic impairment. While we do not yet understand the full spectrum of etiologies, research evidence in animal studies illuminates that persistence of infection is one of them. The persistence of the organism after antibiotic treatment is seen in dogs (5), mice (6, 7), monkeys (8), as well as humans (9), but viable organisms are very difficult to be cultured from the host after antibiotic treatment.

Once the disease has been acquired, it can spread from the skin to various secondary organs throughout the body, including heart, joints, peripheral and central nervous system (10). The early stage of the illness tends to be easier to cure, but it can become more difficult to treat when the disease has progressed to late stage (11). This further challenges conventional antibiotic monotherpy such as intravenous ceftriaxone, which has not been proven successful with a subset of patients presenting with complex chronic symptoms (12).

One of the reasons for this failure, clinically relatable, would be that the host is infected with organisms that are enriched in variant persister forms or the disease when not treated in early stage can progress allowing persisters to further develop (round bodies and biofilm-like microcolonies and larger aggregated biofilm structures). Analogous variant atypical persister forms can be found in stationary phase cultures and under stress conditions such as starvation and antibiotic exposures (13–15). It is worth noting that the current antibiotics used to treat Lyme disease such as Dox, amoxicillin, and cefuroxime (CefU) are highly active against the growing spirochetal form of *B. burgdorferi* but have poor activity against the atypical persister forms (round bodies, microcolonies, and biofilm) enriched in stationary phase cultures (14–17). These persister forms that are not killed by the current Lyme antibiotics may underlie the persistent symptoms in patients despite the standard antibiotic treatment.

To identify drugs that target the persister forms, we screened FDA-approved drug library and NCI compound libraries (14, 18) against stationary phase cultures enriched in round bodies and microcolonies as well as antibiotic-induced round body persisters (19). Using these models, we identified a range of drugs such as daptomycin (Dap), clofazimine, anthracycline antibiotics, and sulfa drugs, etc., which have good activity against the *Borrelia* persister forms. However, some of these persister-active agents are either very expensive, difficult to administer, and have to be given intravenously, or have significant side effects.

Essential oils are concentrated volatile liquids that are extracted from plants, most of which are used as spices and culinary herbs. It has been reported in the literature that essential oils have antimicrobial activities (20), and anecdotal patient reports from the internet suggest that some essential oils may improve symptoms of patients with persistent Lyme disease (http://essentialoiladvocate.info/2015/11/6-essential-oils-to-help-fight-lyme-disease.html/; http://paulaquinlan.com/products/therapeutic-essential-oils/; http://drericz.com/beating-lyme-disease-with-essential-oils/). However, only one study has been done, which assessed the activity of essential oils on *B. burgdorferi*, where it showed that volatile oil from *Cistus creticus* has growth inhibiting activity on growing *B. burgdorferi* (21). Because the current Lyme antibiotics (e.g., Dox, amoxicillin, CefU) are already very good at killing the log phase *B. burgdorferi* but have poor activity against stationary phase *B. burgdorferi* (14, 16, 17). In addition, it is the dormant persister forms enriched in stationary phase cultures (often in variant morphological forms such as round bodies and microcolonies and biofilm) that may be involved in persistent infection that is not cured by the current Lyme antibiotics. Moreover, no study has been performed to evaluate the activity of essential oils on non-growing stationary phase *B. burgdorferi* persisters. Thus, the purpose of this study is to comprehensively evaluate the activity of essential oils for activity against the more difficult to kill persister forms of *B. burgdorferi* that are enriched in the stationary phase culture (22). To achieve this goal, we screened a panel of essential oils from common commercial sources for activities against *B. burgdorferi* stationary phase cells and found that not all essential oils have activity against *B. burgdorferi*, with oregano, cinnamon bark, and clove bud having among the highest anti-persister activity *in vitro*.

## Materials and Methods

### Strain, Media, and Culture Techniques

Low passaged (less than eight passages) *B. burgdorferi* strain B31 5A19 was kindly provided by Dr. Monica Embers (16). The *B. burgdorferi* B31 strain was grown in BSK-H medium (HiMedia Laboratories Pvt. Ltd.) and supplemented with 6% rabbit serum (Sigma-Aldrich, St. Louis, MO, USA). All culture medium was filter-sterilized by 0.2 µm filter. Cultures were incubated in sterile 50 ml conical tubes (BD Biosciences, CA, USA) in microaerophilic incubator (33°C, 5% CO_2_) without antibiotics. After incubation for 7 days, 1 ml stationary-phase *B. burgdorferi* culture (~10^7^ spirochetes per milliliter) was transferred into a 96-well plate for evaluation of potential anti-persister activity of essential oils (see below).

### Essential Oils and Drugs

A panel of commercially available essential oils was purchased from Plant Therapy (ID, USA), Natural Acres (MO, USA), or Plant Guru (NJ, USA). Carvacrol, p-cymene, and α-terpinene were purchased from Sigma-Aldrich (USA). Essential oils were added to BSK-H medium or *B. burgdorferi* cultures to form aqueous emulsion suspensions by vigorous vortexing, followed immediately by serially diluting the essential oil suspensions to desired concentrations into *B. burgdorferi* cultures. Essential oils were also dissolved in organic solvent dimethyl sulfoxide (DMSO) at 20%, followed by dilution at 1:20 into 7-day-old stationary phase culture to 1% final concentration. To make further dilutions for evaluating anti-*Borrelia* activity, the 1% essential oils were further diluted with the stationary phase culture to achieve desired dilutions. Dox, CefU (Sigma-Aldrich, USA), and (Dap) (AK Scientific, Inc., USA) were dissolved in suitable solvents (23, 24) to form 5 mg/ml stock solutions. The antibiotic stocks were filter-sterilized by 0.2 µm filter and stored at −20°C.

### Microscopy

The *B. burgdorferi* cultures were examined using BZ-X710 All-in-One fluorescence microscope (KEYENCE, Inc.). The SYBR Green I/PI viability assay was performed to assess the bacterial viability using the ratio of green/red fluorescence to determine the live:dead cell ratio, respectively, as described previously (14, 22). This residual cell viability reading was confirmed by analyzing three representative images of the bacterial culture using epifluorescence microscopy. BZ-X Analyzer and Image Pro-Plus software were used to quantitatively determine the fluorescence intensity.

### Evaluation of Essential Oils for Their Activities against *B. burgdorferi* Stationary Phase Cultures

To evaluate the activity of essential oils, aliquots of the essential oils or drugs were added to 96-well plate containing 100 µL of the 7-day-old stationary phase *B. burgdorferi* culture to obtain the desired concentrations. In the primary essential oil screen, each essential oil was assayed in four concentrations, 1, 0.5, 0.25, and 0.125% (v/v) in 96-well plate. Dap, Dox, and CefU were used as control drugs at 40, 20, 10, and 5 µM, respectively, since this drug combination has been shown to completely eradicate *B. burgdorferi* persisters in our previous studies (15, 25). The active hits were further confirmed with lower 0.1 and 0.05% concentration; all tests were run in triplicate. All the plates were incubated at 33°C and 5% CO_2_ without shaking for 7 days when the residual viable cells remaining were measured using the SYBR Green I/PI viability assay and epifluorescence microscopy as described (14, 22).

### Antibiotic Susceptibility Testing

To qualitatively determine the effect of essential oils in a high-throughput manner, 10 µl of each essential oil from the prediluted stock was added to 7-day-old stationary phase *B. burgdorferi* culture in the 96-well plate. Plates were sealed and placed in 33°C incubator for 7 days when the SYBR Green I/PI viability assay was used to assess the live and dead cells as described (14). Briefly, 10 µl of SYBR Green I (10,000× stock, Invitrogen) was mixed with 30 µl propidium iodide (PI, 20 mM, Sigma) into 1.0 ml of sterile dH_2_O. Then 10 µl staining mixture was added to each well and mixed thoroughly. The plates were incubated at room temperature in the dark for 15 min followed by plate reading at excitation wavelength at 485 nm and the fluorescence intensity at 535 nm (green emission) and 635 nm (red emission) in microplate reader (HTS 7000 plus Bio Assay Reader, PerkinElmer Inc., USA). With least-square fitting analysis, the regression equation and regression curve of the relationship between percentage of live and dead bacteria as shown in green/red fluorescence ratios was obtained. The regression equation was used to calculate the percentage of live cells in each well of the 96-well plate.

The standard microdilution method was used to determine the MIC of carvacrol, based on inhibition of visible growth of *B. burgdorferi* by microscopy. Carvacrol was added to *B. burgdorferi* cultures (1 × 10^4^ spirochetes per milliliters) to form aqueous suspension by vortex. The carvacrol suspension was twofold diluted from 0.5% (equivalent to 4.88 µg/ml) to 0.008% (equivalent to 0.08 µg/ml). All experiments were run in triplicate. *B. burgdorferi* culture was incubated in 96-well microplate at 33°C for 7 days. Cell proliferation was assessed using the SYBR Green I/PI assay and BZ-X710 All-in-One fluorescence microscope (KEYENCE, Inc.).

### Subculture Studies to Assess Viability of the Essential Oil-Treated *B. burgdorferi* Organisms

A 7-day-old *B. burgdorferi* stationary phase culture (500 µl) was treated with essential oils or control drugs for 7 days in 1.5 ml Eppendorf tubes as described previously (15). After incubation at 33°C for 7 days without shaking, the cells were collected by centrifugation and rinsed with 1 ml fresh BSK-H medium followed by resuspension in 500 µl fresh BSK-H medium without antibiotics. Then 50 µl of cell suspension was transferred to 1 ml fresh BSK-H medium for subculture at 33°C for 20 days. Cell proliferation was assessed using SYBR Green I/PI assay and epifluorescence microscopy as described above.

## Results

### Evaluation of Essential Oils for Activity against Stationary Phase *B. burgdorferi*

**Figure 1:**
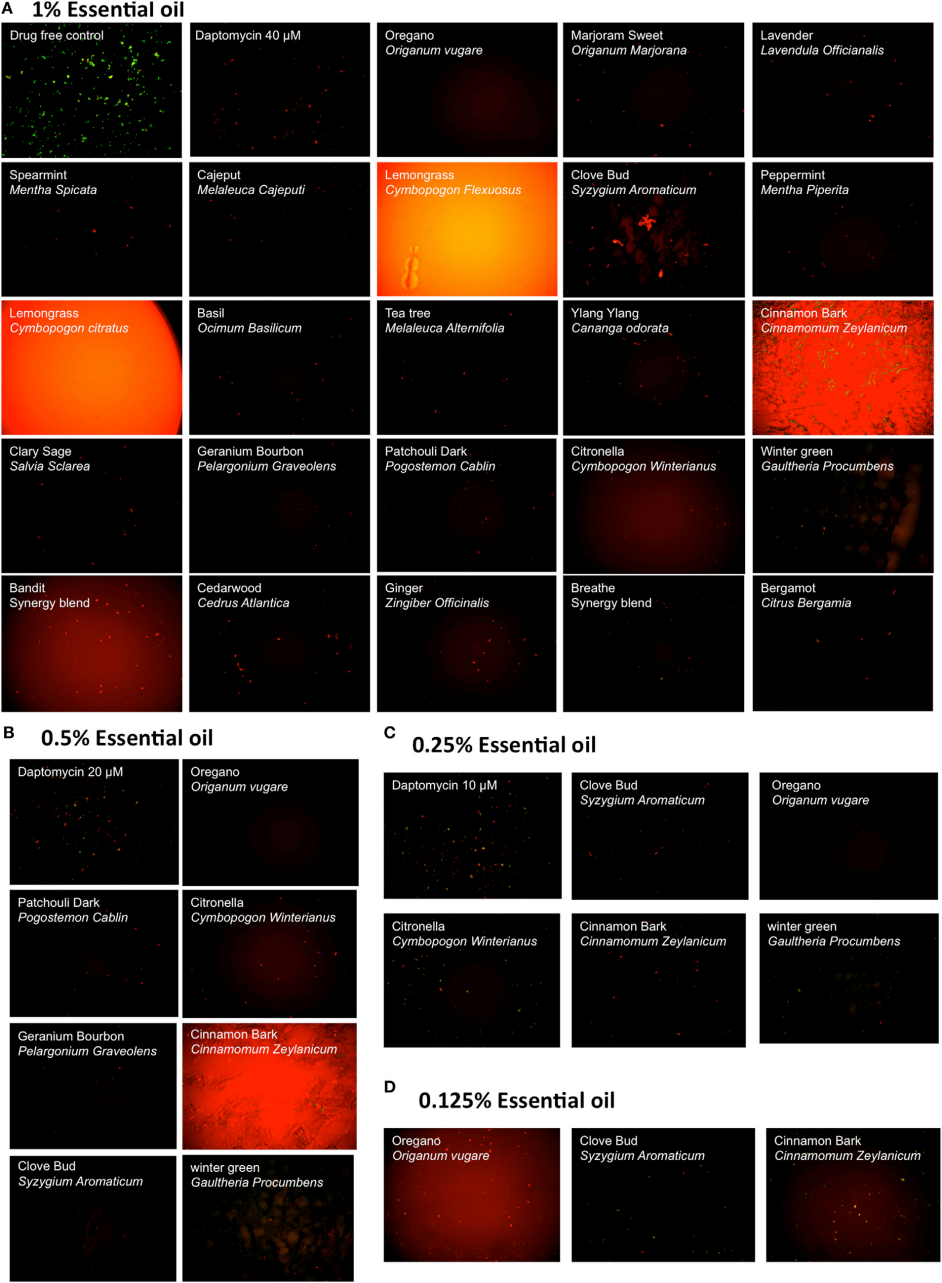
Effect of essential oils on the viability of stationary phase *Borrelia burgdorferi*. A 7-day-old *B. burgdorferi* stationary phase culture was treated with essential oils at different concentrations (v/v), 1% **(A)**, 0.5% **(B)**, 0.25% **(C)**, and 0.125% **(D)** for 7 days followed by staining with SYBR Green I/PI viability assay and fluorescence microscopy. Daptomycin was included as a persister-active positive control drug at 40, 20, and 10 µM in panels **(A–C)**, respectively.

We evaluated a panel of 34 essential oils at four different concentrations (1, 0.5, 0.25, and 0.125%) for activity against a 7-day-old *B. burgdorferi* stationary phase culture in the 96-well plates with control drugs for 7 days. Consistent with our previous studies (14, 25), Dap included as a persister drug control was shown to have higher activity against the *B. burgdorferi* stationary phase culture than the currently used antibiotics such as Dox and CefU for treating Lyme disease (Table 1), with a dose-dependent increase in killing activity. We used 40 µM Dap (64.8 µg/ml) as a positive persister drug control because this is a clinically achievable concentration that could cause near complete clearance of *B. burgdorferi* stationary phase cells while the current Lyme antibiotics could not (14, 15) (Figure 1). Five essential oils (bandit, oregano, clove bud, geranium bourbon, and cinnamon bark) at 1% concentration showed more activity against the stationary phase *B. burgdorferi* culture than 40 µM Dap with the plate reader SYBR green I/PI assay (Table 1). We found some essential oils have autofluorescence, which interfered with the SYBR Green I/PI plate reader assay; however, we were able to resolve this issue present in some samples by fluorescence microscopy. As we previously described (18), we directly calculated the green (live) cell ratio of microscope images using Image Pro-Plus software, which could eliminate the background autofluorescence. Using the SYBR Green I/PI assay and fluorescence microscopy, we additionally found 18 essential oils that showed more or similar activity against the stationary phase *B. burgdorferi* at 1% concentration compared to the 40 µM Dap, included as a positive persister drug control (14), which could eradicate all live cells as shown by red (dead) aggregated cells (Table 1; Figure 1A). At 0.5% concentration, seven essential oils (oregano, cinnamon bark, clove bud, citronella, wintergreen, geranium bourbon, and patchouli dark) were found to have higher or similar activity against the stationary phase *B. burgdorferi* than 40 µM Dap by fluorescence microscope counting after SYBR Green I/PI assay (Table 1; Figure 1B). However, bandit thieves oil, while having good activity at 1%, had significantly less activity at 0.5% and lower concentrations (Table 1). Among the effective hits, five essential oils (oregano, cinnamon bark, clove bud, citronella, and wintergreen) still showed better activity than 40 µM Dap at 0.25% concentration (Table 1; Figure 1C). Eventually, oregano, cinnamon bark, and clove bud were identified as the most active essential oils because of their remarkable activity even at the lowest concentration of 0.125%, which showed similar or better activity than 40 µM Dap (Table 1; Figure 1D).

**Table 1:** Effect of essential oils on a 7-day-old stationary phase *Borrelia Burgdorferi*.^a^

Essential oils and control drugs	Plant	Residual viability (%)^b^			
		1% EO or 40 µM antibiotics	0.5% EO or 20 µM antibiotics	0.25% EO or 10 µM antibiotics	0.125% EO or 5 µM antibiotics
Daptomycin (Dap)		22%	37%	44%	45%
Cefuroxime (CefU)		55%	63%	71%	77%
Doxycycline (Dox)		70%	69%	77%	88%
Oregano	*Origanum vulgare*	**6% (0%)**	**64% (0%)**	**67% (0%)**	**65% (0%)**
Cinnamon Bark	*Cinnamomum zeylanicum*	**16% (ND)^d^**	**18% (ND)^d^**	**21% (0%)**	**36% (24%)**
Clove Bud	*Syzygium aromaticum* L	**6% (0%)**	**24% (0%)**	**22% (0%)**	**39% (20%)**
Citronella^c^	*Cymbopogon winterianus*	**26% (0%)**	**27% (0%)**	**35% (25%)**	79% (66%)
Wintergreen	*Gaultheria procumbens*	**103% (5%)**	**114% (10%)**	**104% (20%)**	104% (70%)
Geranium Bourbon	*Pelargonium graveolens*	**9% (0%)**	**28% (0%)**	41% (66%)	77% (72%)
Patchouli Dark^c^	*Pogostemon cablin*	**26% (0%)**	**55% (0%)**	68% (66%)	76%
Basil	*Ocimum basilicum*	**60% (5%)**	70% (30%)	71% (70%)	76%
Lavender	*Lavendula officianalis*	**27% (0%)**	65% (40%)	70%	78%
Clary Sage	*Salvia sclarea*	**26% (0%)**	70% (45%)	77%	79%
Cedarwood Atlas^c^	*Cedrus atlantica*	**23% (0%)**	69% (47%)	76%	79%
Lemongrass^c^	*Cymbopogon citrates*	**93% (ND)^d^**	77% (48%)	73%	72%
Bandit “Thieves”	Synergy blend	**0 ^e^ (0%)**	40% (50%)	72%	76%
Lemongrass	*Cymbopogon flexuosus*	**67% (ND)^d^**	74% (50%)	72%	82%
Spearmint	*Mentha spicata*	**33% (0%)**	84% (50%)	82%	84%
Tea Tree	*Melaleuca alternifolia*	**31% (0%)**	78% (55%)	81%	76%
Ginger^c^	*Aingiber officinalis*	**65% (0%)**	71% (55%)	71%	77%
Marjoram	*Origanum marjorana*	**22% (0%)**	71% (60%)	74%	76%
Peppermint	*Mentha piperita*	**28% (0%)**	78% (60%)	77%	81%
Bergamot	*Citrus bergamia*	**62% (12%)**	74% (63%)	74%	83%
Breathe	Synergy blend	**32% (18%)**	74% (66%)	74%	74%
Cajeput	*Melaleuca cajeputi*	**36% (0%)**	77% (66%)	75%	76%
Ylang Ylang^c^	*Cananga odorata*	**56% (5%)**	77% (70%)	76%	79%
Anise Star	*Illicium verum hook*	34% (33%)	73%	76%	78%
Stress Relief^c^	Synergy blend	36% (55%)	77%	77%	77%
Cypress^c^	*Cupressus sempervirens*	66%	72%	74%	74%
Orange^c^	*Citrus sinensis*	70%	70%	72%	75%
Eucalyptus	*Eucalyptus globus*	59%	72%	72%	75%
Lemon^c^	*Citrus limonum*	72%	76%	75%	77%
Lime^c^	*Citrus aurantifolia*	73%	76%	75%	77%
Rosemary^c^	*Rosmarinus officinalis*	64%	75%	75%	80%
Pink Grapefruit^c^	*Citrus racemosa*	75%	79%	78%	81%
Tangerine^c^	*Citrus reticulata*	73%	81%	79%	85%
Frankincense^c^	*Boswellia serrata*	81%	85%	94%	94%

*^a^A 7-day-old B. burgdorferi stationary phase culture was treated with essential oils or control drugs Dap, CefU, and Dox for 7 days*.

*^b^Residual viable *B. burgdorferi* was calculated according to the regression equation and ratios of green/red fluorescence obtained by SYBR Green I/PI assay (22). Residual viability calculated by fluorescence microscope is shown in brackets. Bold type indicates the essential oils that had better or similar activity compared with 40 µM Dap used as the active persister-drug control*.

*^c^Essential oils that cannot be dissolved in DMSO*.

*^d^Autofluorescence of essential oil is too strong to be observed under fluorescence microscope*.

*^e^Values are below the 70% isopropanol killed all-dead control*.

**Figure 2:**
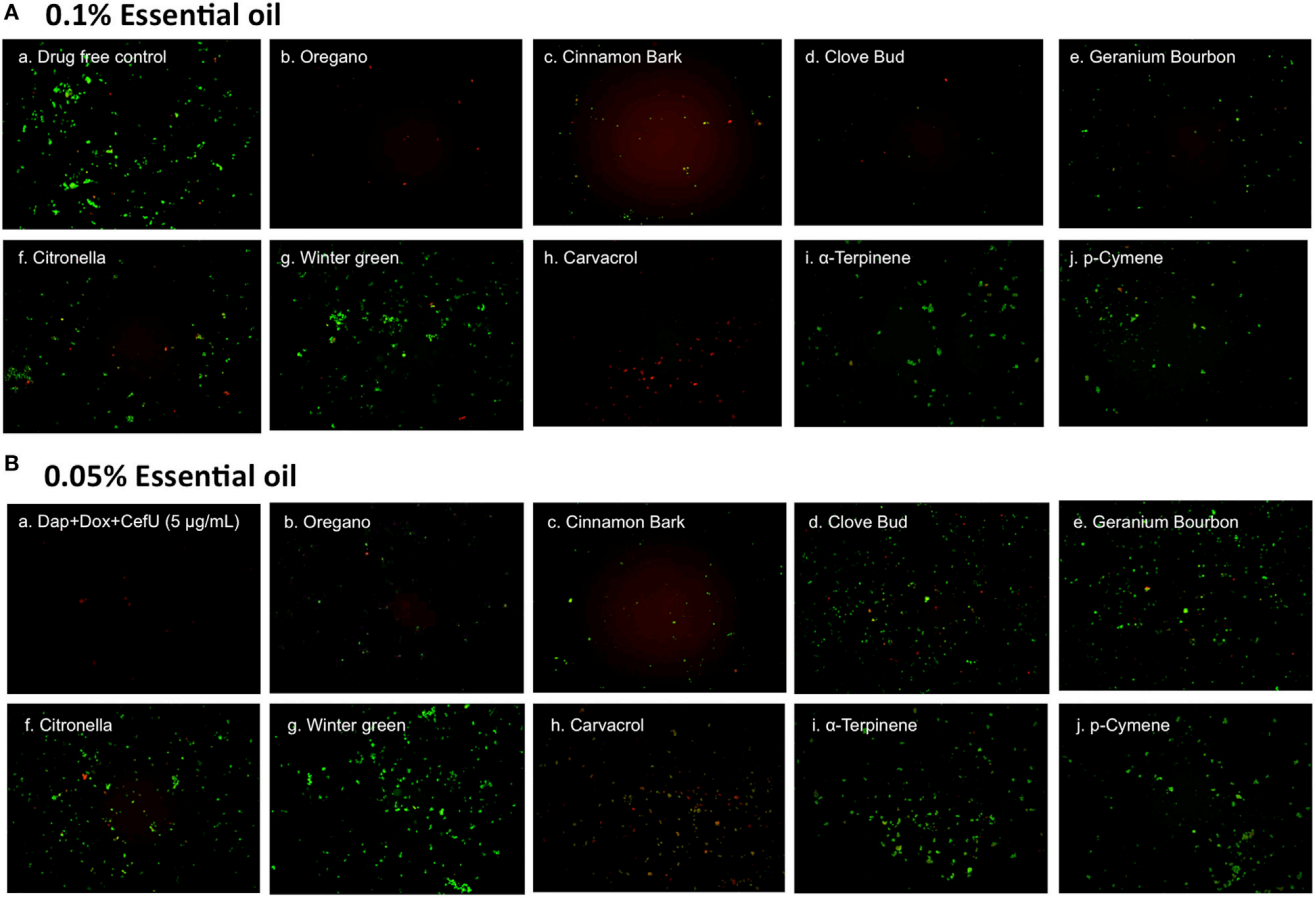
Effect of active essential oils or their ingredients on stationary phase *Borrelia burgdorferi*. A *B. burgdorferi* stationary phase culture (7 days old) was treated with 0.1% **(A)** or 0.05% **(B)** essential oils (labeled on the image) or the ingredients (carvacrol, α-terpinene, or p-cymene) of oregano for 7 days followed by staining with SYBR Green I/PI viability assay and fluorescence microscopy.

To further compare the activity of these active essential oils and find whether they could eradicate stationary phase *B. burgdorferi* at lower concentrations, we evaluated six essential oils (oregano, cinnamon bark, clove bud, citronella, geranium bourbon, and wintergreen) at even lower concentrations at 0.1 and 0.05%. We noticed that oregano could not wipe out stationary phase *B. burgdorferi* at 0.05% concentration as shown by some residual green aggregated cells (Table 2; Figure 2), despite oregano showing strong activity sterilizing all the stationary phase *B. burgdorferi* cells at and above 0.1% concentration (Tables 1 and 2).

**Table 2:** Comparison of essential oil activity against stationary phase *Borrelia burgdorferi* at 0.1 and 0.05% (v/v).^a^

	Residual viability after 0.1% Essential oil treatment		Residual viability after 0.05% Essential oil treatment	
	Treatment^b^	Subculture^c^	Treatment^b^	Subculture^c^
Drug-free control	95%	+	95%	+
Daptomycin + Doxycycline + Cefuroxime^d^	18%^d^	−^d^	N/A	N/A
Oregano	60% (8%)	−	68% (56%)	−
Cinnamon Bark	62% (55%)	−	66% (66%)	−
Clove Bud	57% (33%)	−	68% (77%)	+
Citronella	78% (70%)	+	77% (82%)	+
Geranium Bourbon	74% (70%)	+	85% (80%)	+
Wintergreen	90% (77%)	+	94% (85%)	+
Carvacrol	55% (2%)	−	60% (55%)	−
p-cymene	66% (72%)	+	73% (83%)	+
α-terpinene	70% (77%)	+	77% (85%)	+

*^a^A 7-day-old stationary phase B. burgdorferi was treated with 0.05 or 0.1% essential oils or their ingredients for 7 days when the viability of the residual organisms was assessed by subculture in BSK-H medium*.

*^b^Residual viable percentage of *B. burgdorferi* was calculated according to the regression equation and ratio of green/red fluorescence obtained by SYBR Green I/PI assay as described (14). Direct microscopy counting was employed to rectify the results of the SYBR Green I/PI assay. Residual viability calculated by fluorescence microscopy is shown in brackets. Viabilities are the average of three replicates*.

*^c^“+” indicates growth in subculture; “−” indicates no growth in subculture*.

*^d^Activity was tested with 5 µg/ml of each antibiotic in combination*.

To address potential concern that the essential oils may not dissolve in culture medium well and may affect the above results, we diluted all the 34 essential oils in DMSO and found 19 could be dissolved and 15 could not be dissolved in DMSO (see Table 1, Superscript ^c^). For essential oils that can be dissolved in DMSO, we simultaneously dissolved them in DMSO and also in aqueous culture medium at the same dilutions of 0.5, 0.1, 0.05% and evaluated their activity against the 7-day-old stationary phase *B. burgdorferi*. However, we found that there was no significant difference in their anti-borrelial activity when dissolved in DMSO or in aqueous culture medium (*p* = 0.138–0.975) (see Table S1 in Supplementary Material).

### Carvacrol As a Highly Potent Active Ingredient of Oregano Oil against Stationary Phase *B. burgdorferi*

To identify active ingredients of the oregano essential oil, we tested three major constituents (26), carvacrol, p-cymene, and α-terpinene on the stationary phase *B. burgdorferi*. Interestingly, carvacrol showed similar high activity against *B. burgdorferi* as oregano essential oil either at 0.1% (6.5 µM) or 0.05% (3.2 µM) concentration (Table 2; Figure 2, h). Meanwhile, we also found that carvacrol was very active against replicating *B. burgdorferi*, as shown with a very low MIC of 0.16–0.31 µg/ml. By contrast, p-cymene and α-terpinene did not have activity against the stationary phase *B. burgdorferi* (Table 2; Figure 2, i,j). Thus, carvacrol could be one of the most active ingredients in oregano oil that kill stationary phase *B. burgdorferi*.

### Subculture Studies to Evaluate the Activity of Essential Oils against Stationary Phase *B. burgdorferi*

**Figure 3:**
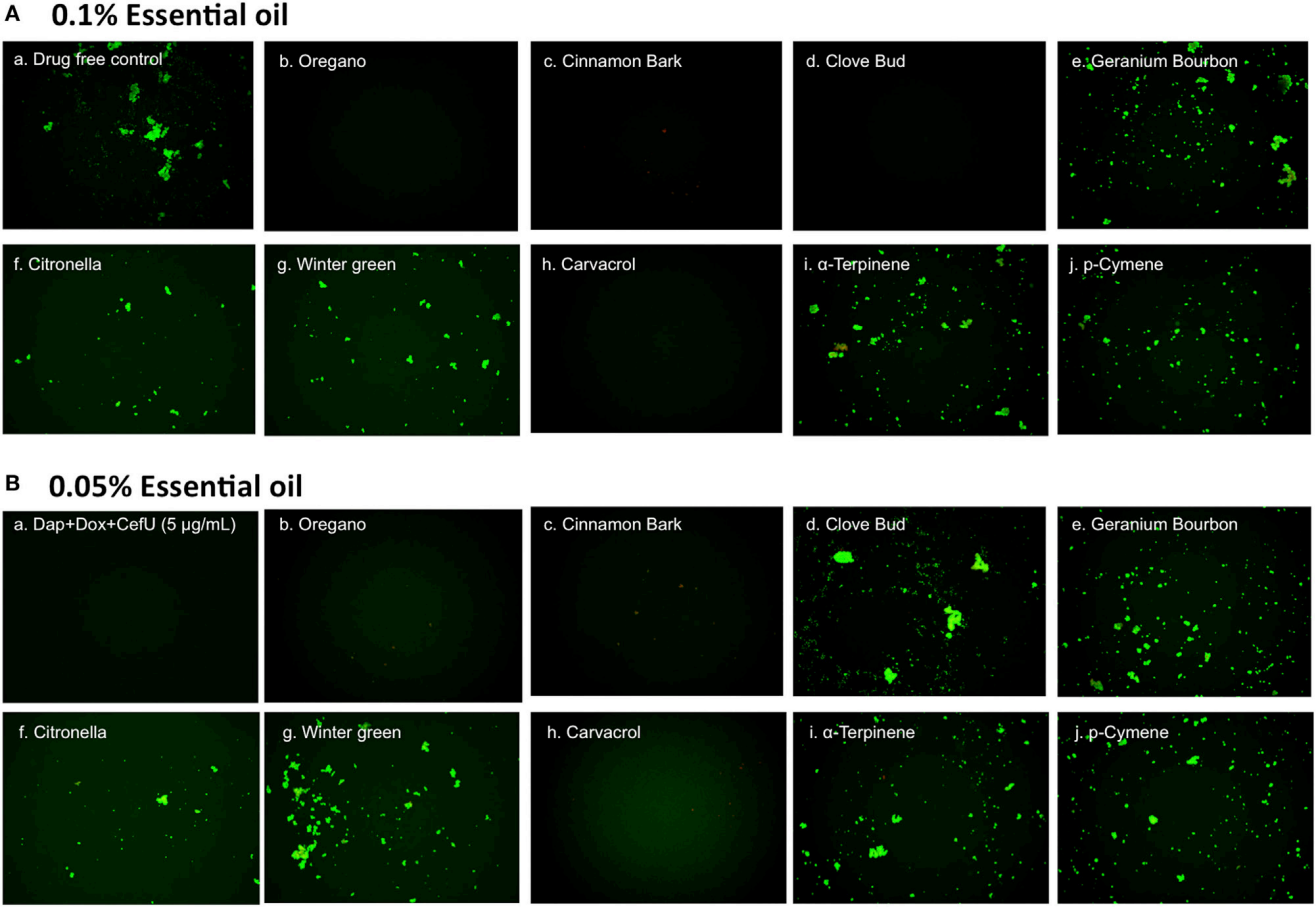
Subculture of *Borrelia burgdorferi* after treatment with essential oils. A *B. burgdorferi* stationary phase culture (7 days old) was treated with the indicated essential oils at 0.1% **(A)** or 0.05% **(B)** for 7 days followed by washing and resuspension in fresh BSK-H medium and subculture for 21 days. The viability of the subculture was examined by SYBR Green I/PI stain and fluorescence microscopy.

To confirm the activity of the essential oils in killing stationary phase *B. burgdorferi*, we performed subculture studies in BSK-H medium as described previously (15). To validate the activity of these essential oils, samples of essential oil-treated cultures were subjected to subculture after removal of the drugs by washing followed by incubation in fresh BSK medium for 21 days. According to the essential oil drug exposure experiments (Table 2), we used subculture to further confirm whether the top six active essential oils (oregano, cinnamon bark, clove bud, citronella, geranium bourbon, and wintergreen) could eradicate the stationary phase *B. burgdorferi* cells at 0.1 or 0.05% concentration. At 0.1% concentration, the subculture results were consistent with the above drug exposure results, and no regrowth in samples of three top hits, oregano, cinnamon bark, and clove bud was observed (Figure 3A, b–d). However, citronella, geranium bourbon, and wintergreen could not completely kill the stationary phase *B. burgdorferi* with many spirochetes being visible after 21-day subculture (Figure 3A, e–g). Subculture also confirmed the activity of carvacrol by showing no spirochetal regrowth in the 0.1% carvacrol-treated samples. However, in the p-cymene and α-terpinene subculture samples, growth in 0.1% concentration samples was observed. At 0.05% concentration, we observed no spirochetal regrowth after 21-day subculture in the oregano and cinnamon bark-treated samples (Figure 3B, b,c), even though some very tiny aggregated microcolonies were found after treatment (Figure 2B, b,c). Although the clove bud showed better activity than the cinnamon bark at 0.05% concentration (Table 2), it could not sterilize the *B. burgdorferi* stationary phase culture, as they all had visible spirochetes growing after 21-day subculture (Figure 3B, c,d). Additionally, 0.05% citronella, geranium bourbon, and wintergreen could not kill all stationary phase *B. burgdorferi* since many viable spirochetes were observed in the 21-day subculture (Figure 3B, e–g). Remarkably, 0.05% carvacrol sterilized the *B. burgdorferi* stationary phase culture as shown by no regrowth after 21-day subculture (Figure 3B, h).

## Discussion

Previous *in vitro* studies showed that certain essential oils have antibacterial activity against multidrug resistant Gram-negative clinical isolates (27). In this study, we tested 34 essential oils from different plants on non-growing stationary phase *B. burgdorferi* as a model of persister drug screens. We were able to identify 23 essential oils at 1% concentration that are more active than the control persister drug Dap (40 µM or 64.8 µg/ml) 3 of which, oregano, clove bud, and cinnamon bark, highlighted themselves as having a remarkable activity even at a very low concentration of 0.125% (Table 1). Among them, oregano and cinnamon bark essential oils demonstrated the best activity as shown by complete eradication of stationary phase *B. burgdorferi* even at 0.05% concentration. In a previous study, oregano oil was found to have antibacterial activity against Gram-positive and Gram-negative bacteria (26). Here, for the first time, we identified oregano oil as having a highly potent activity against stationary phase *B. burgdorferi*. We tested three major ingredients of oregano oil (carvacrol, p-cymene, and α-terpinene) on *B. burgdorferi*, and found carvacrol is the major active component, which showed similar activity as the complete oregano oil (Figures 2 and 3). We could not rule out the possibility that other components may also have activity against *B. burgdorferi*. We plan to use GC/MS to identify components of the active essential oils and test them on *B. burgdorferi* in the future.

In addition to the above findings, we noted that oregano oil can dramatically reduce the size of aggregated biofilm-like microcolonies compared to the antibiotic controls (Figure 1). After treatment with 0.25% oregano essential oil, only some dispersed tiny red aggregated cells were left in the culture (Figure 1C). Interestingly, we observed that amount and size of aggregated biofilm-like microcolonies of *B. burgdorferi* dramatically reduced with increasing concentrations of oregano oil, as aggregated biofilm-like structures vanished after treatment with 0.5 or 1% oregano essential oil. When we reduced the concentration of oregano essential oil to 0.05%, it could not eradicate stationary phase *B. burgdorferi* (residual viability 56%, Figure 2B, b) but the size of aggregated microcolonies decreased significantly. By contrast, Dap could kill the aggregated biofilm-like microcolonies of *B. burgdorferi* as shown by red aggregated microcolonies but could not break up the aggregated microcolonies even at the highest concentration of 40 µM (Figure 1A). It has been shown that carvacrol and other active compositions of oregano oil could disrupt microbial cell membranes (20). Future studies are needed to determine whether oregano oil and other active essential oils have similar membrane disruption activity and could destroy the aggregated biofilm-like structures of *B. burgdorferi*.

We also noted that some essential oils such as oregano and cinnamon bark had relatively high residual viability percentage (Table 2) at a low concentration of 0.05%, but the treated *B. burgdorferi* cells did not grow in the subculture experiment (Table 2; Figure 3B, b,c). We speculate that these essential oils could dissolve the dead *B. burgdorferi* cells presumably due to their high lipophilicity. The reduction of the number of dead red cells by the essential oil made the residual viability percentage increase, although the number of live cells obviously decreased as well (Figure 2A, b–d and Figure 2B, b,c). In addition, these essential oils may also permanently damage *B. burgdorferi* during the treatment, such that even in the fresh medium, the residual *B. burgdorferi* cells still could not regrow.

Meanwhile, we found that, at a high concentration (above 1%), lemongrass or oregano essential oil showed apparent high residual viability percentage by the SYBR Green I/PI plate assay, compared with the microscopy counting data (Table 1; Figure 1A). This may be caused by strong autofluorescence of these essential oils that severely interfere with the SYBR Green I/PI assay. We studied the emission spectra of lemongrass essential oil using Synergy H1 multi-mode reader and found lemongrass essential oil emits the strongest autofluorescence. The peak fluorescence of lemongrass essential oil is at 520 nm that overlaps with the green fluorescence of SYBR Green I dye (peak is at 535 nm). The strong autofluorescence caused the abnormal residual viability percentage (above 100% in Table 1) using SYBR Green I/PI plate assay. We also found that the oregano essential oil emits autofluorescence at 535 nm, which pushed the green/red fluorescence ratio higher than their true values (Table 1). We were able to solve this problem by using fluorescence microscopy as a more reliable measure to confirm the results of SYBR Green I/PI plate reader assay (14, 18).

Additionally, we found cinnamon bark and clove bud essential oils showed excellent activity against stationary phase *B. burgdorferi*. Cinnamon bark essential oil eradicated the stationary phase *B. burgdorferi* cells even at 0.05% concentration (Table 2) while clove bud essential oil showed sterilization at 0.1% or above concentration. Extractions of cinnamon bark and clove bud have been used as flavors for food processing. Based on this discovery, effective oral regimens with low side effect may be developed to fight against Lyme disease in future studies.

In a previous study, it has been found that volatile oil from *C. creticus* showed growth inhibiting activity against growing *B. burgdorferi in vitro* (21), but its activity against stationary phase bacteria enriched in persisters was not evaluated. In this study, we tested six *Citrus* plants (*Citrus bergamia, Citrus sinensis, Citrus limonum, Citrus aurantifolia, Citrus racemosa, Citrus reticulata*) on the stationary phase *B. burgdorferi* culture and found bergamot (*C. bergamia*) had high activity (residual viability 12%) at 1% concentration. The other *Citrus* essential oils did not show good activity against *B. burgdorferi* compared with clinically used Dox, CefU, or Dap (Table 1).

Although we found several essential oils (oregano, cinnamon bark, clove bud) that have excellent activity against *B. burgdorferi* stationary phase cells *in vitro* (Table 1), the effective dose that will show equivalent activity *in vivo* is unknown at this time largely because the active ingredients in the active essential oils and the pharmacokinetic profile of the active ingredients are not all known. Future studies are needed to identify the active ingredients of the active essential oils and determine their effective dosage *in vivo*. Identification of active components or active component combinations from essential oils may help to eliminate the quality difference of natural products such that MICs and MBCs of the active compounds could be tested on growing bacteria in future studies. We were able to identify carvacrol as the most active ingredient in oregano essential oil, and its pharmacokinetics has been studied as a feed addition in pigs (28) and topical oil in cattle (29). In the rat model, the calculated LD50 of carvacrol is 471.2 mg/kg (30). We noticed that the 0.05% of carvacrol used here, which is equivalent to 0.48 µg/ml or 3.2 µM and completely eradicated *B. burgdorferi* stationary phase cells in subculture (Figure 3), is lower than the peak plasma concentration (3.65 µg/ml) in the swine study (28). Clinically, an antimicrobial agent that penetrates the blood–brain barrier as well as the persistent *Borrelia* organisms do, would be an ideal candidate for further study (31). Oil of Oregano is a good candidate for this consideration given that its main active ingredient—carvacrol is a phenolic monoterpenoid that is fat-soluble and found in animal studies to have blood–brain barrier penetration (32). These findings favor the application of carvacrol in future treatment studies. Importantly, carvacrol seems to be more active than Dap, the most active persister drug against *B. burgdorferi* (14, 15). In this study, 0.1% carvacrol (6.4 µM) showed much higher activity (2% residual viability) than 5 µM Dap (45% residual viability) (Tables 1 and 2). In addition, 0.05% carvacrol (3.2 µM) could eradicate *B. burgdorferi* stationary phase cells with no regrowth in subculture, by contrast, 10 µg/ml Dap (6.2 µM), could not completely kill *B. burgdorferi* stationary phase cells as shown by regrowth in subculture (15). Furthermore, we know that the drug’s activity against non-replicating bacteria is not always consistent with its activity against growing bacteria. With that in mind, we also tested carvacrol on the growing *B. burgdorferi* cells and found its MIC to be 0.16–0.31 µg/ml. This result showed that carvacrol is a good drug candidate active against not only stationary phase *B. burgdorferi* but also log phase replicating cells. For consideration in clinical applications, there is limited safety information on carvacrol and essential oils in humans. In mice, carvacrol has been given at 40 mg/kg daily for 20 days with no apparent toxicity (33). However, carvacrol and other active components of essential oils showed certain cytotoxicity (IC_50_ of carvacrol was 200–425 µM) (34, 35) on mammalian cells and genotoxic activity *in vivo* (10 mg/kg) (36). We found that few essential oils have been used in human studies. This is probably because these essential oils are natural products, which are mixtures of multiple active components, and little is known about their active ingredients or their mechanisms of antibacterial action. Thus, more work is needed to identify their active compounds of the effective essential oils. In addition, it is well known that some effective drugs identified *in vitro* may fail when tested *in vivo*. Thus, adequate animal studies are needed to confirm the safety and efficacy of the active essential oils in *in vivo* setting before human studies.

In summary, we found that essential oils had varying degrees of activity against stationary phase *B. burgdorferi*. The most active essential oils are oregano, cinnamon bark, and clove bud, which seem to have even higher activity than the persister drug Dap. A particularly interesting observation is that these highly active essential oils had remarkable biofilm-dissolving capability and completely eradicated all stationary phase cells with no regrowth *in vitro*. In addition, carvacrol was found to be the most active ingredient of oregano with high activity against *B. burgdorferi* stationary phase cells. Future studies are needed to test whether carvacrol could replace the persister drug Dap in drug combinations against more resistant biofilm-like structures and for treating persistent *Borrelia* infections in animal models and in patients.

## Author Contributions

YZ and JM conceived the experiments; JF, WS, and SZ performed the experiments; JF, NZ, JM, and YZ analyzed the data; and JF, NZ, JM, and YZ wrote the paper.

## Conflict of Interest Statement

The authors declare that the research was conducted in the absence of any commercial or financial relationships that could be construed as a potential conflict of interest. The reviewer AN and handling editor declared their shared affiliations.
