# Supplementary material for: Selective Essential Oils from Spice or Culinary Herbs Have High Activity against Stationary Phase and Biofilm Borrelia burgdorferi
Source: Front Med (Lausanne). 2017 Oct 11;4:169. doi: 10.3389/fmed.2017.00169 (PMC5641543; doi:10.3389/fmed.2017.00169)
Supplement: Supplementary file 1 [file Table_1.DOCX]

Table S1. Comparison of essential oil activity dissolved in DMSO versus aqueous medium.

|  |  | **Oregano** | | **Cinnamon Bark** | | **Clove Bud** | | **Geranium Bourbon** | |
| --- | --- | --- | --- | --- | --- | --- | --- | --- | --- |
|  |  | **Viability%** | **Standard deviation** | **Viability%** | **Standard deviation** | **Viability%** | **Standard deviation** | **Viability%** | **Standard deviation** |
| 0.5% essential oil | DMSO stock | 65% | 1.6% | 19% | 1.3% | 25% | 0.7% | 33% | 1.6% |
|  | Aqueous suspension | 63% | 1.7% | 20% | 0.9% | 26% | 2.4% | 31% | 0.7% |
|  | *p-*value | 0.283 | | 0.802 | | 0.532 | | 0.260 | |
| 0.1% essential oil | DMSO stock | 68% | 2.1% | 63% | 2.3% | 44% | 2.5% | 76% | 2.0% |
|  | Aqueous suspension | 66% | 1.6% | 62% | 1.1% | 46% | 0.8% | 76% | 1.4% |
|  | *p-*value | 0.361 | | 0.556 | | 0.305 | | 0.806 | |
| 0.05% essential oil | DMSO stock | 64% | 1.4% | 67% | 2.4% | 64% | 1.4% | 80% | 2.4% |
|  | Aqueous suspension | 64% | 1.2% | 65% | 1.1% | 65% | 1.7% | 80% | 2.0% |
|  | *p-*value | 0.955 | | 0.138 | | 0.474 | | 0.975 | |
